# A Molecular Insight into the Role of Antioxidants in Nonalcoholic Fatty Liver Diseases

**DOI:** 10.1155/2022/9233650

**Published:** 2022-05-11

**Authors:** Devaraj Ezhilarasan, Thangavelu Lakshmi

**Affiliations:** ^1^Department of Pharmacology, Molecular Medicine and Toxicology Lab, Saveetha Dental College, Saveetha Institute of Medical and Technical Sciences (SIMATS), 600 077, Chennai, Tamil Nadu, India; ^2^Department of Pharmacology, Mandy Dental College, University of Dhaka, Bangladesh

## Abstract

Nonalcoholic fatty liver disease (NAFLD) defines fat accumulation in the liver, and it is commonly associated with metabolic syndromes like diabetes and obesity. Progressive NAFLD leads to nonalcoholic steatohepatitis (NASH) and ultimately causes cirrhosis and hepatocellular carcinoma, and NASH is currently a frequent cause of liver transplantation. Oxidative stress is often contributed to the progression of NAFLD, and hence, antioxidants such as silymarin, silybin, or silibinin, pentoxifylline, resveratrol, and vitamins A, C, and E are used in clinical trials against NAFLD. Silymarin induces the peroxisome proliferator-activated receptor *α* (PPAR*α*), a fatty acid sensor, which promotes the transcription of genes that are required for the enzymes involved in lipid oxidation in hepatocytes. Silybin inhibits sterol regulatory element-binding protein 1 and carbohydrate response element-binding protein to downregulate the expression of genes responsible for de novo lipogenesis by activating AMP-activated protein kinase phosphorylation. Pentoxifylline inhibits TNF-*α* expression and endoplasmic reticulum stress-mediated inflammatory nuclear factor kappa B (NF-*κ*B) activation. Thus, it prevents NAFLD to NASH progression. Resveratrol inhibits methylation at Nrf-2 promoters and NF-*κ*B activity via SIRT1 activation in NAFLD conditions. However, clinically, resveratrol has not shown promising beneficial effects. Vitamin C is beneficial in NAFLD patients. Vitamin E is not effectively regressing hepatic fibrosis. Hence, its combination with antifibrotic agents is used as an adjuvant to produce a synergistic antifibrotic effect. However, to date, none of these antioxidants have been used as a definite therapeutic agent in NAFLD patients. Further, these antioxidants should be studied in NAFLD patients with larger populations and multiple endpoints in the future.

## 1. Introduction

Nonalcoholic fatty liver disease (NAFLD) describes a fatty liver condition (hepatic steatosis) with or without hepatic injury [1]. Globally, NAFLD affects 25 to 30% of the general population and the Middle East and South America have the highest and Africa has the lowest prevalence of NAFLD [2, 3]. In the majority of patients, NAFLD is nonprogressive, and a few patients' have progressive fat accumulation and hepatic injury lead to inflammation and fibrosis, a condition termed nonalcoholic steatohepatitis (NASH) [4]. In some individuals, NASH progresses to cirrhosis and its complications with further decompensation leading to death or liver transplantation [5, 6]. The existence of metabolic syndromes, including obesity, insulin resistance/diabetes, dyslipidemia, systemic hypertension, and hyperglycemia, are commonly associated risk factors for NAFLD and NASH. Bidirectionally, NAFLD may augment numerous features and comorbidities of metabolic syndromes [7]. Among different metabolic syndromes, diabetes mellitus shows the strongest biological link to NAFLD progression; clinically, up to 75% of type 2 diabetes patients have NAFLD [8, 9]. Approximately, 50% of hypertensive patients have NAFLD and it is associated with pathological features such as arterial stiffness, myocardial hypertrophy and dysfunction, heart failure, and kidney disease [10–12]. The incidence of NASH to hepatocellular carcinoma (HCC) progression rate is high (~35–50%) in individuals with type 2 diabetes, obesity, and older age [13, 14]. Thus, repurposing antidiabetic and antiobesity medicine is also considered one of the major treatment options for NAFLD and NASH [15]. It has been predicted that NAFLD will be one of the major chronic liver diseases responsible for liver transplantation in adults and children in this decade [16]. There is no specific noninvasive method available for NASH detection [17]. To date, ultrasound is the best way of diagnosing early fatty liver and liver biopsy remains a gold standard method for diagnosing NAFLD [18]. Thus, NAFLD has emerged as a major challenge due to its prevalence, diagnostic difficulties, the complexity of its pathogenesis, and lack of specific treatments. Presently, no specific treatment is approved for NAFLD; however, weight loss by lifestyle modification, including avoiding red meat intake, low fiber food, trans fats, carbohydrates, and a high-fructose diet along with daily physical workouts, is certainly helpful to regress the NAFLD. The available treatment modalities for NAFLD include medical therapies, bariatric surgery, and endoscopic bariatric interventions [19].

### 1.1. Pathogenesis of NAFLD

**Figure 1:**
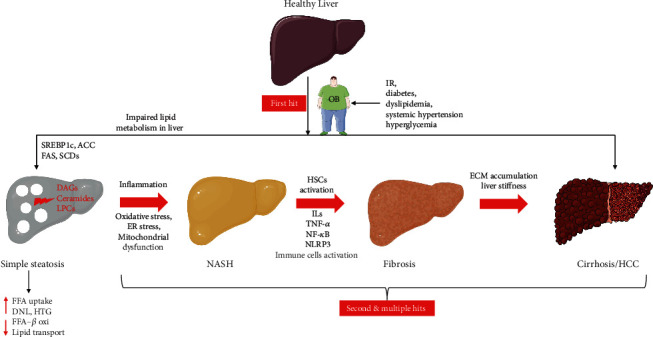
Pathological progression of nonalcoholic fatty liver diseases (NAFLD) and nonalcoholic steatohepatitis (NASH) to hepatocellular carcinoma (HCC). IR: insulin resistance; OB: obesity; SREBP1c: sterol regulatory element-binding transcription factor 1c; ACC: acetyl-CoA carboxylase; FAS: fatty acid synthase; SCDs: stearoyl-CoA desaturase; DAGs: diacylglycerols; LPCs: phosphatidylcholines; FFA: free fatty acids; DNL: de novo lipogenesis; HTG: hepatic triglyceride; *β*-oxi: *β*-oxidation; ER: endoplasmic reticulum; HSCs: hepatic stellate cells; ILs: interleukins; TNF-*α*: tumor necrosis factor-*α*; NF-*κ*B: nuclear factor-kappa B; NLRP3: nucleotide-binding domain leucine-rich repeat (NLR) and pyrin domain containing receptor 3; ECM: extracellular matrix.

In the majority of patients, hepatic steatosis development is commonly associated with intake of high dietary fat [20]. NAFLD is pathologically characterized by the presence of excessive fat accumulation (≥5%) in the liver in the absence of viral infection, alcohol consumption, or lipotoxic drugs [21]. Histologically, NAFLD was classified based on the percentage of lipid accumulation in hepatocytes as mild (5% to 33%), moderate (34% to 66%), and severe (>66%) [22]). NAFLD occurs due to increased free fatty acids import and de novo hepatic lipogenesis [20]. Dietary sugars contain glucose and fructose converted into fatty acids in the liver via de novo lipogenesis. Sterol regulatory element-binding proteins (SREBP) 1c, transcription factor, promote de novo lipogenesis by regulating the expression of acetyl-coenzyme A carboxylase 1, fatty acid synthetase (FAS), and stearoyl-coenzyme A desaturase enzymes involved in lipogenesis. SREBP-1 remains activated in NAFLD, thus deteriorating steatosis. Dietary fat converts triglyceride (TG) into fatty acids in adipose tissue, and it is released into circulation and enters the liver. In the liver, fatty acids can be oxidized by mitochondria or transformed again into TG for export into the circulation as VLDL. Impairment in the above homeostasis leads to the accumulation of fatty acids, which promotes the generation of lipotoxic species such as diacylglycerols, ceramides, and lysophosphatidyl choline that mediate mitochondrial dysfunction, endoplasmic reticulum (ER) stress, hepatocellular injury, inflammation, and cell death by apoptosis to produce NASH [16]). Apart from aberrant metabolism, bacterial endotoxin released from the gut and other sources is also implicated in the aggravation of NAFLD to NASH [23]. The pattern of the onset of NASH was classically put forth by Day and James [24]. The NAFLD-based lipotoxicity can be explained by their “double-hit” hypothesis. In the “first hit,” increased intracellular TG accumulation and steatosis occur due to insulin resistance cause by hepatic de novo lipogenesis and impairment in fatty acid export. During the “second hit,” NAFLD progresses into NASH by elevation of intracellular reactive oxygen species (ROS) that increase oxidative stress to facilitate inflammation and cell death. Further, the “third hit”/“multiple hit” hypotheses were also reported, in which excessive oxidative stress induces cell death, reduces the mature hepatocyte replication, and results in liver cirrhosis and HCC [25]. Together, lipid accumulation occurs in the “first hit,” which increases endoplasmic reticulum (ER) stress, mitochondrial dysfunction, and intracellular oxidative stress and decreases endogenous antioxidant synthesis in the “second hit.” Hepatocellular ballooning, necrosis, and toxicity occur in the “third hit.” Thus, excessive TG and free fatty acids in the liver induce lipotoxicity and oxidative stress, leading to inflammation, fibrosis, and cell death [26]. Though NAFLD and NASH are multifactorial diseases, oxidative stress is responsible for the initiation of liver injury in NAFLD and its subsequent progression into NASH, fibrosis, cirrhosis, and HCC (Figure 1).

### 1.2. Oxidative Stress and NAFLD

Oxidative stress plays a key role in the initiation of NAFLD and its progression into NASH. As mentioned above, disturbance in lipid metabolism leads to fat accumulation in hepatocytes, which triggers intracellular organelles such as mitochondria, endoplasmic reticulum, and NADPH oxidase to generate ROS. The increased mitochondrial fatty acid oxidation stimulates ROS generation within the electron transport chain components (I, II, and III) upstream of cytochrome c oxidase. Particularly, increased *β*-oxidation of fatty acids in mitochondria and microsomes seems to produce more ROS in NAFLD [27]. Mitochondria generate ATP via oxidative phosphorylation, and superoxide radicles are also generated as the byproduct of oxidative phosphorylation. Similarly, NADPH oxidase and ER stress alterations also contribute to oxidative stress in NAFLD [28]. Oxidative stress commonly occurs intracellularly when there is an imbalance between the levels of intracellular ROS and endogenous enzymic and nonenzymic antioxidants (Ezhilarasan,[29]. Clinically, decreased endogenous antioxidants have been reported in NAFLD patients [30]. In clinical subjects, superoxide dismutase (SOD), catalase (CAT), reduced glutathione (GSH), glutathione peroxidase (GPx), and GSH reductase (GR) in serum/plasma have been reportedly modulating in early and advanced NAFLD patients [31]. Increased intracellular ROS induces changes in insulin sensitivity and the modulation of several crucial enzymes responsible for lipid metabolism. In hepatic steatosis, oxidative stress triggers immune cell responses [32]. The experimental and clinical studies have demonstrated the infiltration of adaptive immune cells (T cells) in the liver during NASH and the presence of circulating antibodies directed toward antigens taking origin from oxidative stress [33, 32]. Thus, redox signaling and innate immune signaling interactions form a complex network that regulates inflammatory responses. In NAFLD, oxidative stress induces the activation of numerous redox-sensitive transcription factors (nuclear factor kappa B (NF-*κ*B), early growth response-1, and activator protein 1) and proinflammatory mediators (tumor necrosis factor *α* (TNF-*α*), interleukins (IL), etc.) leading to liver inflammation, fibrosis, and cell death [34–36].

Mechanistically, NAFLD is a multifactorial disease condition involving oxidative stress, insulin resistance and binge intake of fat, and a carbohydrate-based diet that causes accumulation of excessive fat in the liver leading to steatosis [37]. Simple liver steatosis can cause intracellular ROS upregulation via CYP2E1 induction. The increased intracellular ROS, in turn, causes oxidative stress [38]. The accumulations of fat, ROS, and concomitant intracellular antioxidants decrease, causing lipotoxicity, mitochondrial dysfunction, and ER stress in the liver. Fatty infiltration in hepatocytes leads to impairment in *β*-oxidation and oxidative phosphorylation in mitochondria, impaired *β*-oxidation peroxisome, and lysosome dysfunction result in the intracellular ROS and hydrogen peroxide radicals' accumulation [39]. Thus, impaired lipid metabolism was implicated in the alteration of oxidant and antioxidant homeostasis that causes redox imbalance and oxidative stress. The impaired lipid metabolism in hepatocytes increases the fatty acid uptake via CD36, and mitochondrial dysfunction can cause the accumulation of intracellular triglycerides. Redox imbalance in the fatty liver increases ER stress by upregulation of unfolded protein response. The chronic ER stress and sustained unfolded protein response activation increase the expressions of ER stress proteins such as PKR-like ER kinase, activating transcription factor 4, 6, and CCAAT-enhancer-binding protein homologous protein, leading to activation of proinflammatory marker expression and cell death pathways in hepatocytes [40]. Further, sustained ER stress leads to the activation of sterol regulatory element-binding protein 1C (SREBP1c). Its nuclear translocation can cause the transcription of lipogenesis-associated genes. Thus, oxidative stress is playing a major role in the initiation and aggravation of NAFLD.

### 1.3. Lifestyle, Diet, and NAFLD

A sedentary lifestyle with less physical activity and poor diet is often associated with NAFLD occurrence [41]. Therefore, lifestyle modifications and diet restrictions are considered noninterventional and primary therapy for NAFLD [17]. For instance, weight reduction of up to 5–10% caused 58%-90% of NASH and 45% of fibrosis resolution [42, 43]. Dietary habits are one of the common etiological factors associated with the development and severity of NAFLD [44]. A recent meta-analysis reported that individuals who consume a high amount of red meat and soft drinks might be more likely to develop NAFLD. This study also suggests that consumption of fruits, vegetables, whole and refined grains, dairy products, eggs, fish, and legumes had no significant influence on NAFLD development. Interestingly, higher nut intake was negatively associated with NAFLD [45]. Mediterranean diet generally contains low carbohydrate, rich in antioxidants and anti-inflammatory agents including carotenoids, polyphenols, fiber, polyunsaturated fats, and vitamins [46]. Clinical studies have shown that the Mediterranean diet is beneficial in preventing risk factors for cardiovascular diseases and metabolic syndromes like NAFLD [47, 48]. Mediterranean diet with polyphenol and antioxidant-rich green plants and less red meat or processed meat consumption was shown to double intrahepatic fat loss [49]. Generally, antioxidants have cytoprotective effects by nullifying excessive intracellular free radical-induced oxidative stress. On the other hand, oxidative stress has been implicated in the pathological progression of NAFLD. Therefore, several natural and synthetic antioxidants have been previously evaluated against experimental and clinical NAFLD conditions.

### 1.4. Antioxidants and NAFLD

**Figure 2:**
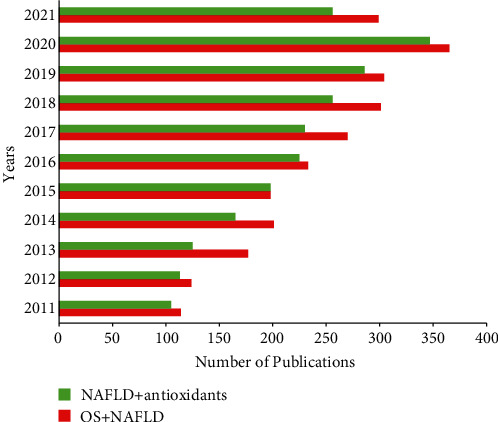
Publication index on oxidative stress and NAFLD and antioxidant treatments in NAFLD in the last 10 years. The red bar indicates the number of research articles published on oxidative stress-induced NAFLD. The green bar indicates the number of research articles published on antioxidant interventions in oxidative stress-induced NAFLD. Data source: PubMed/Medline.

As aforementioned, increased lipid peroxidation and decreased antioxidant status have been associated with the NAFLD progression. Thus, oxidative stress is often contributed to NAFLD progression, and hence, several antioxidants have been studied experimentally and clinically against NAFLD patients [50]. In the last decade, several clinical and experimental studies have implicated oxidative stress in NAFLD conditions and targeted NAFLD with antioxidants (Figure 2). This review focuses on the effect of some of the well-studied plant-derived and synthetic antioxidants and antioxidant vitamins that are previously studied against experimental and clinical NAFLD conditions. For instance, antioxidants such as silymarin, silybin, or silibinin, pentoxifylline, resveratrol, and vitamins A, C, and E have reached clinical trials against NAFLD. Therefore, the following sections deal with the efficacy of these antioxidants that are evaluated against experimental and clinical NAFLD conditions.

## 2. Antioxidants in Experimentally Induced NAFLD

### 2.1. Silymarin and Silybin

Silymarin, a standard hepatoprotective agent isolated from the seeds of milk thistle, has been used to treat various liver ailments [51]. Experimental studies have reported that silymarin can protect the liver from oxidative stress, inflammation, steatosis, fibrosis, and HCC [52–54]. Silymarin at a 200 mg/kg dose significantly reduced the fructose-induced NAFLD by decreasing ER stress proteins such as glucose regulatory protein 78 and X-box-binding protein 1 (Sahin et al., [55]. Silymarin attenuated the high-fat diet- (HFD) induced oxidative stress and decreased high-density lipoprotein cholesterol (HDL-C), low-density lipoprotein cholesterol (LDL-C), and hepatic TG levels in NAFLD mice. Further, silymarin treatments reduced the mRNA expression of enzymes responsible for de novo lipogenesis such as SREBP1c, FAS, and acetyl-CoA carboxylase 1 in diabetic obese mice with NAFLD. NAFLD-induced NADPH oxidase components such as p40phox, p47phox, and p67phox and enzymic antioxidants (SOD and CAT) were decreased after silymarin administration in NAFLD mice, indicating the antioxidant and hypolipidemic effect [56]. Silymarin also reduced fructose diet-induced oxidative stress, dyslipidemia, and steatosis in NAFLD rats [57]. Silymarin has poor aqueous soluble properties, and hence, its membrane permeability and oral bioavailability are low [58]. Therefore, silymarin-loaded lipid polymer hybrid nanoparticles containing chitosan are synthesized to increase the bioavailability and therapeutic efficacy against NAFLD. The chitosan-based silymarin nanopreparation has 14.38-fold higher oral bioavailability than the conventional silymarin preparations, and its administration reduced macrovesicular steatosis in NAFLD mice (Liang et al., [59]. Silymarin was also shown to be effective against NASH induced in juvenile C57Bl/6 mice fed with HFD immediately after weaning. This study suggests that silymarin containing HFD administration for 12 weeks was found effective in the absence of changes in the dietary habits in a juvenile model of NASH (Marin et al., [60]).

Silybin or silibinin (50 or 100 mg/kg/day), an active ingredient of silymarin, treatments for four weeks, significantly reduced serum and liver fat accumulation in HFD-induced NAFLD in mice. Serum and liver metabolomic analysis revealed that silybin could reverse HFD-induced metabolic disorders in mice [61]. Peroxisome proliferator-activated receptor *α* (PPAR*α*) is increasingly expressed in the liver, and it regulates the expression of genes responsible for gluconeogenesis, fatty acid oxidation, and lipid transport [62]. Therefore, PPAR*α* agonists are previously studied against HFD-induced NAFLD in experimental models and clinical models (Yoo et al., [63, 64]. A study conducted by Zhu et al. [65] has shown the antioxidant, anti-inflammatory, and lipid-lowering effect of *Silybum marianum* oil in HFD-induced NAFLD in mice. Silybin was proven as a PPAR*α* partial agonist, and therefore, the presence of silybin in *Silybum marianum* oil can be attributed to the lipid-lowering effect of PPAR*α* activation. In this line, a recent study has also shown that silybin can ameliorate the methionine-choline-deficient (MCD) diet-induced NAFLD in mice, and the lipid-lowering effect of silybin was correlated via PPAR*α* activation (Cui et al., [66]). Silybin attenuates the MCD caused oxidative stress and decreases oxidative stress-mediated lipid accumulation and inflammation. The oxidative stress was overcome by promoting the upregulation of Nrf2 target genes, and inflammation was suppressed by inhibiting the proinflammatory mediator release and NF-*κ*B signaling by silybin in NASH mice (Ou et al., [67]. Silibinin restores nicotinamide adenine dinucleotide^+^ (NAD^+^), a coenzyme involved in redox reactions, levels by inhibiting poly(ADP-ribose) polymerase and activates the SIRT1/AMP-activated protein kinase (AMPK) pathway *in vitro* and *in vivo* (Salomone et al., [68]. Lower AMPK activity was associated with de novo lipogenesis in NAFLD (von Loeffelholz et al., [69]. Therefore, silybin-induced AMPK activation can be related to decreased de novo lipogenesis. The anti-inflammatory effect of silybin was achieved by SIRT2 activity. Interestingly, supplementing NAD^+^ with silybin was useful to maintain SIRT2 activity. Silybin was shown to inhibit ER stress and NLRP3 inflammasome activation in HFD-fed NAFLD mice (Zhang et al., [70]).

### 2.2. Pentoxifylline (PTX)

TNF-*α* is one of the main pathological drivers that initiate liver injury, inflammation, and NAFLD to NASH progression (Kakino et al., [71]). Mice deficient in TNF receptors exhibit reduced lipid accumulation, inflammation, and fibrosis in the experimental NASH model (Tomita et al., [72]). Clinically, increased TNF-*α* levels were reported in the serum of NAFLD patients (Hui et al., [73]. Pharmacological TNF-*α* receptor inhibition by antibodies and specific inhibitors attenuates TNF-*α*-mediated liver inflammation and reduces steatosis and fibrosis in NAFLD (Wandrer et al., [74]. Pentoxifylline was shown to inhibit TNF-*α* synthesis (Ghasemnejad-Berenji et al., [75]. In experimental studies, in rats, 8 weeks of PTX treatment reduced HFD-induced oxidative stress, transaminases elevation, insulin resistance, and inflammation via TNF-*α* inhibition (Zaitone et al., [76]). In NAFLD mice with concurrent type 2 diabetes, PTX administration reduced steatosis and hyperglycemia by inducing fatty acid *β*-oxidation (Ye et al., [77]. In a rodent model of NASH-related dimethylnitrosamine-induced hepatocarcinogenesis, PTX administration reduced serum and hepatic TG content, serum transaminases, and fatty acids. Further, PTX treatments also reduced the mRNA expression of proinflammatory markers and lipid metabolism markers such as FAS, SREBP-1c, stearoyl-CoA desaturase-1, and carnitine palmitoyltransferase 1A in the NASH-related liver preneoplasms model (Shirakami et al., [78]. In MCD diet-fed rats, intraperitoneal PTX administration reduced ER stress and TNF-*α*-mediated inflammation and NASH. PTX administration significantly downregulated ER stress-associated protein (GRP78, phosphorylation of eukaryotic initiation factor-2*α*, activating transcription factor 4 and 6, inositol-requiring enzyme 1, and CCAAT-enhancer-binding protein homologous protein expression in NAFLD rats) (Chae et al., [79]). These ER stress proteins increase intracellular ROS and also activate the NF-*κ*B-mediated inflammatory signaling (Liu and Green, [80] responsible for NAFLD to NASH progression in the steatotic liver. Thus, it could be stated that the downregulation of ER stress and related protein expression by PTX can be attributed to its anti-inflammatory effect. However, in a Guinea pig-fed HFD-induced NAFLD model, pentoxifylline treatment for 8 weeks did not reduce steatosis, inflammation, and fibrosis (Ipsen et al., [81]. Surprisingly, PTX treatment (100 mg/kg) for 4 days/week for three weeks was shown to exacerbate fatty liver in obese and diabetic ob/ob mice by increasing intestinal glucose absorption and activating hepatic lipogenesis and it was suggested that PTX could aggravate fatty liver in patients with preexisting hyperglycemia (Massart et al., [82]).

### 2.3. Antioxidant Vitamins in NAFLD

Vitamins regulate various key enzymatic processes in the liver, and alterations in vitamin metabolism are reported to play a crucial role in NAFLD progression. Vitamins A, C, and E are well-studied against NAFLD due to their antioxidant activities. Similarly, modulation of vitamins D and B_12_ and folate levels in serum also had a strong correlation with NAFLD severity [83]. Hepatic stellate cells (HSC) store most of the body's retinol [84]. However, impaired vitamin A metabolism caused its accumulation in the hepatocytes rather than HSC of NAFLD mice. Thus, NAFLD causes vitamin A accumulation in hepatocytes, which may cause disease progression [85]. Retinoic acid treatments have been shown to offer antioxidant effects by decreasing mitochondrial ROS and SOD2 upregulation in mice. Retinoic acid treatment also increased Sirt1 hepatic expression and inhibited SREBP1c expression in HFD-fed WT mice *in vivo* and *in vitro* [86]. Vitamin C supplementation reduces the fatty acid burden in the liver by promoting the gene expression of PPAR*α*-dependent fatty acid *β*-oxidation genes in HFD-induced mice [87]. Interestingly, prophylactic administration of vitamin C (15 and 30 mg/kg/day) has significantly reduced the body weight and steatosis, thereby decreasing NAFLD risk in mice. In a therapeutic study, 30 mg/kg/day of vitamin C administration attenuated steatosis and NAFLD in mice. However, prophylactic administration of a high dose of vitamin C (90 mg/kg/day) did not reduce the risk of NAFLD development. In fact, a high dose of vitamin C administration significantly increased body weight, adipose tissue mass, and inflammation [88]. This study clearly shows that vitamin C dose should be fixed in NAFLD conditions. In choline-deficient diet-induced NAFLD rats, vitamin C (30 mg/kg/day) administration significantly inhibited oxidative stress and steatosis in NAFLD rats. At the same time, vitamin E (200 mg/day) administration was not found effective [89]. In a Guinea pig, MCD diet-induced NASH model, megadose (2.5 g/kg/day) of vitamin C administration reduced macrovesicular steatosis; however, AST and ALT increased even after vitamin C administration [90].

In contrast, a recent study has reported that vitamin C deficiency leads to the inhibition of NAFLD. Vitamin C-deficient senescence marker protein 30 knockout mice had reduced NAFLD progression than wild-type mice. Vitamin C-deficient mice had increased levels of carbohydrate responsive element-binding protein and SREBP-1c and decreased FAS expression [91], suggesting long-term vitamin C deficiency may be useful to inhibit de novo lipogenesis through impaired sterol regulatory element-binding protein-1c activation. However, vitamin C deficiency-mediated NAFLD inhibition has to be elaborately studied. In HFD induced with phosphatidylethanolamine N-methyltransferase-deficient NAFLD mice, 3 weeks of vitamin E administration (0.5 g/kg) normalized cholesterol metabolism and also reduced oxidative stress-related inflammation and fibrosis; however, it failed to decrease hepatic TG content [92]. Vitamin E administration attenuated fructose diet-induced NAFLD by activation of the Nrf2/carboxylesterase 1 signaling pathway involved in the lipogenesis [93]. Vitamin E also reduced partial hepatectomy-induced NAFLD in mice by attenuating oxidative stress [94]. Therefore, as an antioxidant, vitamin E was widely studied as an adjuvant agent along with other drugs against NAFLD conditions.

## 3. Resveratrol

**Figure 3:**
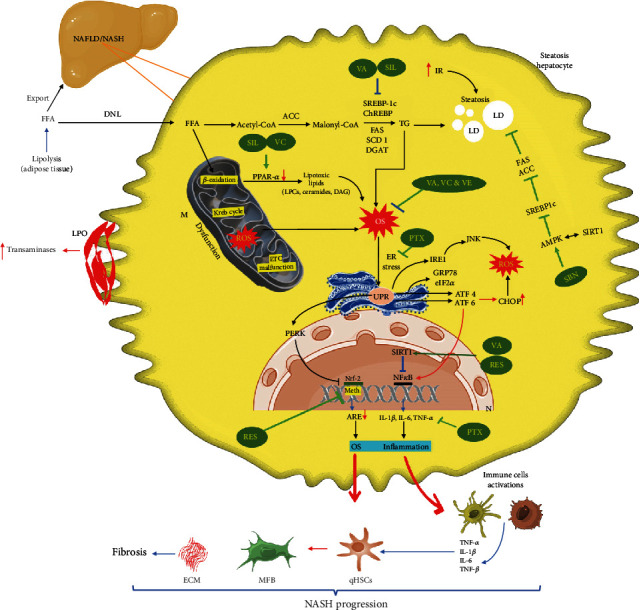
Molecular mechanisms of antioxidant-mediated nonalcoholic fatty liver disease (NAFLD)/nonalcoholic steatohepatitis (NASH) regression. SIL: silymarin; SBN: silibinin/silybin; VA: vitamin A; VC: vitamin C; VE: vitamin E; RES: resveratrol; PTX: pentoxifylline; LPO: lipid peroxidation; FFA: free fatty acids; DNL: de novo lipogenesis; DGAT: diglyceride acyltransferase; LD: lipid droplets; M: mitochondria; N: nucleus; ETC: electron transport chain; PPAR-*α*: peroxisome proliferator-activated receptor-alpha; TG: triglyceride; IR: insulin resistance; SREBP1c: sterol regulatory element-binding transcription factor 1c; ACC: acetyl-CoA carboxylase; FAS: fatty acid synthase; SCDs: stearoyl-CoA desaturase; DAGs: diacylglycerols; ER: endoplasmic reticulum; qHSCs: quiescent hepatic stellate cells; ILs: interleukins; TNF-*α*: tumor necrosis factor-*α*; NF-*κ*B: nuclear factor-kappa B; TGF-*β*: transforming growth factor-*β*; ECM: extracellular matrix; OS: oxidative stress; Meth: methylation; NRF-2: nuclear factor erythroid 2-related factor 2; ARE: antioxidant response element; UPR: unfolded protein response; PERK: PKR-like ER kinase; AMPK: 5′ AMP-activated protein kinase; GRP78: glucose-regulated protein 78; eIF2*α*: phosphorylation of eukaryotic initiation factor-2*α*; ATF 4 and 6: activating transcription factor 4 and 6; IRE1: inositol-requiring enzyme 1; CHOP: CCAAT-enhancer-binding protein homologous protein; ROS: reactive oxygen species; JNK: c-Jun N-terminal kinase.

Resveratrol is a plant-derived polyphenol and antioxidant used in experimental and clinical NAFLD conditions [95]. In *in vitro* study, resveratrol treatments reversed fatty acid mixtures (oleic or palmitic acid) induced mitochondrial oxidative stress-mediated steatosis in HepG2 cells [96]. In NASH mice, resveratrol treatment decreases oxidative stress and upregulates antioxidants and lipolytic enzymes by SIRT1 activation [97]. Resveratrol treatments reduced hepatic TG levels and decreased expression of FAS, and SREBP-1c was associated with HFD-induced methylation of the Nrf2 promoter in the mouse liver. Resveratrol treatment also decreased high-glucose-induced ROS and methylation of the Nrf2 promoter in HepG2 cells [98]. These findings suggest that resveratrol may attenuate NAFLD by epigenetic modulation. Resveratrol is considered a potent AMPK activator [99]) at a molecular level; resveratrol increases AMPK phosphorylation and decreases SREBP-1c, responsible for lipogenesis and lipid accumulation, by liver X receptor inhibition [100]. Besides, resveratrol, a known SIRT 1 activator, inhibits autophagy-mediated liver inflammation (Choi et al., [100]). Resveratrol treatments improved lipid metabolism, redox homeostasis, and oxidative stress in HFD-induced NAFLD in rats via the protein kinase A/AMPK/PPAR*α* signaling pathway (Huang et al., [101]). Gut microbiota dysbiosis has been often linked with the existence of obesity and diabetes with NAFLD (Aron-Wisnewsky et al.,[102]). Resveratrol treatment failed to attenuate high-fat and fructose-induced dysbiosis in the gut microbiota (Milton-Laskibar et al., [103]). However, a recent report found that resveratrol could improve liver steatosis and IR in NAFLD by significantly changing the diversity and gut microbiota composition (Du et al., [104]). These studies show that resveratrol may have a beneficial effect on NAFLD via AMPK and SIRT 1 activation. The molecular mechanisms behind antioxidant-induced modulation of NAFLD are presented in Figure 3.

## 4. Antioxidants Used in Clinical Trial for NAFLD

### 4.1. Silymarin and Silybin

Silymarin is one of the well-studied plant-derived antixoidant compounds in NAFLD patients. In an RCT on 99 patients, compared to placebo (*n* = 50), silymarin treatment (700 mg, *n* = 49) thrice daily for 48 weeks has significantly reduced AST to platelet ratio and fibrosis score and improved liver histology without inducing silymarin-related side effects (Wah Kheong et al., [105]). In a phase 2 clinical trial (NCT00680407) in NASH patients with no cirrhosis history, silymarin (Legalon®) treatment (420 and 700 mg) for 48 weeks did not show significant histological improvement (Navarro et al., [106]). Clinical studies show that silymarin treatments for 6 months can lower transaminases and GGT activities in serum and hepatorenal brightness ratio, and inflammatory markers (not significantly) are also reduced in NAFLD patients (Cacciapuoti et al., [107]). Meta-analysis has shown that silymarin is beneficial in reducing transaminase activity in NAFLD patients compared to placebo, irrespective of weight loss (Solhi et al., [108]; Zhong et al., [109]; Kalopitas et al., [110]). Silymarin has the potential to regress NAFLD in patients under the Mediterranean diet than in the Mediterranean diet alone group. For instance, along with the Mediterranean diet, twice-daily administration of silymarin (210 mg) for 6 months has led to significant improvement in the glycemic profile and regression of liver damage (Colletta et al., [111]). Silymarin, vitamin C, vitamin E, and coenzyme Q10 and selenomethionine (Medronys epato®) contain a capsule twice a day for 45- and 90-days treatments that has histologically improved liver function and reduced the marker enzymes of liver toxicity (ALT, AST, ALP, and GGT) and lipid markers (total cholesterol, high-density lipoprotein cholesterol (HDL-C), low-density lipoprotein cholesterol (LDL-C), and TG) in serum of NAFLD patients (Curcio et al., [112]). In another study, treatment with silymarin (Eurosil 85®) and *α*-tocopherol (MEDAS SL) along with a low-calorie diet (1520 kcal) with exercise for three months significantly reduced ALT and AST activities and improved liver function in NAFLD patients (Aller et al., [113]). A six-month administration of silybin with vitamin D and vitamin E (RealSIL 100D®) improved oxidative stress, inflammation, and fibrosis in NAFLD patients (Federico et al., [114]). In an RCT, the hepatoprotective effect of a natural antioxidant cocktail containing silymarin, curcumin, docosahexaenoic acid, choline, and *α*-tocopherol was studied against NAFLD patients. This antioxidant cocktail treatment was well tolerated and decreased the transaminases and GGT from 23.2 to 3.7%; however, there was no significant change observed in the metabolic and inflammatory marks in NAFLD patients (Cerletti et al., [115]).

### 4.2. Pentoxifylline

An RCT in NASH patients showed that PTX is beneficial to reduce the transaminases, insulin resistance, and adiponectin levels, and there were no significant changes that were reported in the metabolic markers in these patients (Sharma et al., [116]). PTX treatment for 400 mg/thrice daily for one year only improves hepatic histological activity but not lobular inflammation, ballooning, and fibrosis in NASH patients (Alam et al., [117]). PTX (1200 mg/day, three divided doses) was shown beneficial along with fenofibrate (300 mg/day) for 24 weeks resulting in reduced liver fibrosis and stiffness, insulin resistance, and inflammation in NAFLD patients (El-Haggar et al., [118]). PTX administration (400 mg/thrice daily) and metformin (500 mg/thrice daily) for six months in NASH patients improved insulin resistance and reduced transaminase levels and cardiovascular risk (Ćulafić et al., [119]). A meta-analysis using 5 RCT of 147 patients with NAFLD/NASH reported that PTX treatment could reduce body weight, glucose, liver transaminases, and TNF-*α* level. PTX also improved lobular inflammation, steatotic grade, ballooning, and fibrosis (Du et al., [120]). PTX and pioglitazone were found equally effective in patients with NAFLD and newly detected glucose intolerance (Karim et al., [121]). Therefore, PTX can be used as an adjuvant along with the drugs used for metabolic diseases.

### 4.3. Antioxidant Vitamins

Serum levels of vitamins A, C, and E are reportedly decreasing in NAFLD patients with advanced liver fibrosis (Coelho et al., [122]). The antioxidant vitamins certainly modulate liver diseases; vitamins A, C, D, and E have promising therapeutic potential that can influence NAFLD management (Abe et al., [123]). Therefore, NAFLD patients are targeted with dietary intervention strategies with vitamins A, C, and E. In a cross-sectional study of a large cohort of NAFLD subjects, vitamins C and E (1000 kcal) intake decreased the odds of NASH (Ivancovsky-Wajcman et al., [124]). Vitamin E treatment (200-800 IU/d) for 24 weeks caused a ≥25% relative decrease in intrahepatic TG level in NAFLD patients (*n* = 20) (Podszun et al., [125]). Vitamins C (500 mg/day) and E (800 mg/day) combination treatment for one year has reduced oxidative stress damage, steatosis, and fibrosis scores in nondiabetic NASH patients (Barbakadze et al., [126]). In a cross-sectional study in middle-aged and older adults, a moderate inverse association was reported between dietary vitamin C intake (≥146.07 mg/day) and NAFLD (Wei et al., [127]).

In a recent RCT, vitamin C (1000 mg/day) supplementation for 12 weeks profoundly improved plasma vitamin C, adiponectin, liver disease, and glucose metabolism in NAFLD patients (He et al., [128]). A systematic review and meta-analysis confirmed that there was no histological improvement in pediatric NAFLD after vitamin E intake (Sarkhy et al., [129]). Retinoic acid, an active vitamin A metabolite, and its receptor (retinoic X receptor *α*) mRNA levels in serum are inversely related to steatosis (intrahepatic TG) and severity of NAFLD. The retinoic acid levels were significantly lower in NAFLD (1.42 ± 0.47 ng/mL) and NASH (1.14 ± 0.26 ng/mL) compared to the normal subjects (2.70 ± 0.52 ng/mL) (Liu et al., [130]). Dietary vitamins A and C intake has partly improved SOD levels and protected NAFLD in patients (Ma et al., [131]).

### 4.4. Resveratrol

Resveratrol shows a beneficial effect against experimentally induced NAFLD. Clinically, along with lifestyle modification, resveratrol supplementation (500 mg) for 12 weeks significantly reduced liver inflammation and hepatic fibrosis in NAFLD patients (Faghihzadeh et al., [58]). A study reported by the same group shows that resveratrol treatments effectively reduced liver fibrosis and lipid markers and were not effective in reducing cardiovascular risk in NAFLD patients (Faghihzadeh et al., [132]). Clinical studies also report that resveratrol does not effectively reduce oxidative stress in NAFLD patients (Asghari et al., [133]). However, a systematic review and a meta-analysis revealed that resveratrol supplementation is ineffective in NAFLD patients with liver fibrosis (Elgebaly et al., [134]). A recent meta-analysis by Rafiee et al. [135] also reported that resveratrol supplementation only improves inflammatory markers and it is not beneficial for the management of NAFLD. The antioxidants that underwent clinical trials against NAFLD and NASH patients are reported in Table 1.

### 4.5. Challenges and Future Perspectives

Several studies have shown that silymarin is well tolerated and it could reduce transaminases activity in NAFLD patients. Therefore, silymarin can be used in NAFLD patients with elevated liver transaminases. Silymarin is well studied clinically; however, silybin has not been studied elaborately in clinical subjects. Silybin has also been shown to possess antioxidant, lipid-lowering, and anti-inflammatory effects in experimentally induced NAFLD and NASH. Besides, silybin is a partial PPAR*α* agonist, and therefore, it could be beneficial to modulate the beta-oxidation of fats and transportation of lipids from the liver. PTX was shown to be beneficial against experimental NAFLD. However, some experimental studies have also shown the ineffectiveness of PTX in NAFLD, and Massart et al. [82] have shown that PTX could activate hepatic lipogenesis and aggravate preexisting hyperglycemia in mice. PTX underwent clinical trials and a meta-analysis reports that PTX treatment is beneficial in NAFLD patients. PTX at 1200 mg/day in three divided doses is well tolerated in NAFLD patients. However, most of the studies have reported the ineffectiveness of PTX on steatosis and inflammation in NASH conditions. However, PTX is more effective when given along with the drugs used in metabolic disorders, including metformin, fenofibrate, and pioglitazone. Identifying natural SIRT1 and AMPK agonists would certainly help to reduce steatosis progression by downregulation of enzymes responsible for lipogenesis. Though experimental studies report beneficial effects of resveratrol, clinically, it is not effective in NAFLD subjects. Vitamin C is reported to be beneficial in NAFLD patients. Long-term administration of vitamin C seems to be beneficial, and therefore, future clinical studies are warranted with a larger population and multiple endpoints.

Though phytochemicals have been reported to have beneficial effects against a variety of ailments, their poor bioavailability hinders their efficacy. The low bioavailability of herbal medicine has been associated with several factors like poor absorption, interaction with food, phase I and II biotransformation, and gut microbiota (Di Lorenzo et al.,[140]. Flavonoids have larger molecular weight and complex structures; their bioavailability is generally low due to their poor solubility, poor permeability, and poor stability (Thilakarathna and Rupasinghe, [141]. Dietary factors like fat intake were shown to improve the bioavailability of flavonoid compounds (Gonzales et al., [142]), while protein intake decreased their bioavailability (Swieca et al., [143]). The gut microbiota plays a critical a role in flavonoid absorption and metabolism. Gut microbiota hydrolyzes flavonols, flavones, isoflavones, and anthocyanins into their respective lipophilic aglycones before their intestinal absorption. Flavonoids undergo extensive first-pass metabolism via phase II methylation, sulfation, and glucuronidation biotransformation reactions in the liver after their absorption from the gut (Lotito et al., [144]). Flavonoid conjugation reactions produce glucuronides and sulfate derivatives enabling their excretion via urine and bile (Thilakarathna and Rupasinghe, [141]. Thus, the complex nature of flavonoids, poor solubility and stability, presence of food, gut microbiota, and extensive intestinal and first-pass metabolisms in the liver affect the bioavailability of a variety of flavonoid compounds. Therefore, to enhance the oral bioavailability of flavonoids, nanodrug delivery systems have been previously used in the form of nanoparticle emulsions, self-emulsifying systems, and solid dispersions, and these studies came out with promising results (Yang et al., [145]).

Like low bioavailability and nonspecific selectivity of flavonoids generally hampers their therapeutic efficacy in clinical settings. In general, flavonoids are complex molecules shown to modulate several signaling pathways associated with several diseases. For instance, most of the flavonoid compounds are shown to exhibit cytotoxic effect via intracellular ROS accumulation in cancer cells (Thakur and Devaraj, [146]; Elumalai et al., [147]). However, flavonoid compounds such as apigenin, eriodictyol, 3-hydroxyflavone, kaempferol, luteolin, naringenin, quercetin, rutin, and taxifolin exhibited cytotoxicity via abnormal ROS level in human lung embryonic fibroblasts (TIG-1) and human umbilical vein endothelial (HUVE) cells (Matsuo et al., [148]). This study suggests that flavonoid compounds can induce ROS signaling for cytotoxicity in normal cells. Thus, flavonoid compounds used as anticancer medicines might be toxic to normal cells of the body. Further, the off-target effects of flavonoid compounds need to be elaborated further.

## 5. Conclusion

Undoubtedly, NAFLD is the most common form of chronic liver disease worldwide and it is strongly linked with the presence of oxidative stress, mitochondrial dysfunction, and inflammation. *Silybum marianum* plant products, especially silybin, have a specific role in modulating oxidative stress and lipid metabolism. The antioxidant effect of silybin was achieved by activation of Nrf-2-related genes, and the lipid-lowering effect was achieved by promoting the PPAR*α*, and the anti-inflammatory effect was achieved by the inhibition of the NF-*κ*B signaling. Resveratrol, a known SIRT 1 and AMPK activator, inhibits the SREBP1c responsible for de novo lipogenesis. Resveratrol also inhibits methylation at Nrf-2 promotor and protects NAFLD liver from epigenetic alterations. Long-term vitamin C is reported to be beneficial by improving adiponectin and reducing hepatic TG level, odds of NASH in NAFLD patients. However, the beneficial effect of the antioxidant vitamins in NAFLD patients remains inconclusive.

## Figures and Tables

**(a):** 

Antioxidants	NAFLD condition	Study type	Status/outcome	Trial number/reference
Alpha tocopherol plus ascorbic acid	Inflammation, fibrosis, insulin resistance	Phase 2 and 3	Diet and physical exercise are effective in NAFLD children compared to antioxidant therapy	NCT00655018 (Bugianesi et al., [136])
Tocovid Suprabio 200 mg (tocotrienols/vitamin E)	Hepatic steatosis NAFLD	Phase 2	NA	NCT04704063
Omega 3 fatty acids	Fatty liver	Interventional (clinical trial)	NA	NCT04281121
Omega-3 fatty acid (DHA EE) and vitamin E	NAFLD and NASH	Phase 2	Undergoing	NCT04198805
Metadoxine	NAFLD and prediabetes	Phase 4	NA	NCT02051842
Lovaza (omega-3-acid ethyl esters)	NAFLD	Phase 4	NA	NCT00941642
Hydroxytyrosol plus vitamin E	NAFLD	Phase 3	NA	NCT02842567
Vitamin E	Fatty liver	Phase 2	No association with the reduction of bodyweight and improvement in insulin sensitivity. Intrahepatic TG reduced by 27%	NCT01792115 (Podszun et al., [125])
Vitamin E	NAFLD and NASH	Phase 2	NA	NCT03669133
Vitamin E	NAFLD and NASH	NA	NA	NCT02690792

**(b):** 

Antioxidants	NAFLD condition	Study type	Status/outcome	Trial number/reference
Siliphos-selenium-methionine-alpha lipoic acid	NASH	Phase 4	NA	NCT01650181
Legalon® 140 mg (silymarin)	NAFLD	NA	New (not started yet)	NCT05051527
Silymarin	NAFLD and HCV	Phase 1	NA	NCT00389376
Silymarin and ornithine aspartate granule	NAFLD	Phase 4	Undergoing	NCT05042245
Silymarin	NAFLD	NA	NA	NCT03749070
Silymarin	NAFLD	Phase 2	Reduced AST to platelet ratio, fibrosis score, and improved liver histology	NCT02006498 (Wah Kheong et al., [105])
Resveratrol	NAFLD, type 2 diabetes, metabolic syndrome	Phase 2 and 3	NA	NCT02216552
Resveratrol	NAFLD and obesity	NA	NA	NCT01446276
Resveratrol	Fatty liver	NA	NA	NCT01464801
Purified anthocyanin	NAFLD	Phase 1	NA	NCT01940263
Alpha lipoic acid	NAFLD	Phase 4	Undergoing	NCT04475276
S-Adenosyl-L-methionine (SAMe)	NAFLD and NASH	Phase 3	NA	NCT01754714

**(c):** 

Antioxidants	NAFLD condition	Study type	Status/outcome	Trial number/reference
Pentoxifylline (PTX)	NASH	Phase 2	PTX significantly improved steatosis, lobular inflammation, and liver fibrosis	NCT00590161 (Zein et al., [137])
PTX	Fatty liver diseases	Phase 2	NA	NCT02283710
PTX	NASH	Phase 2 and 3	PTX is safe and well tolerated. In NASH patients, PTX improves histology; PTX failed to reduce transaminases	NCT00267670 (Van Wagner et al., [138])
PTX and vitamin E	NASH	Phase 3	Combinational therapy regressed fibrosis compared to vitamin E alone	NCT01384578 (Kedarisetty et al., [139])
PTX, vitamin E, and ursodeoxycholic acid	NASH	Phase 4	NA	NCT04977661
PTX and SAMe	NASH	NA	NA	NCT02231333

NA: not available. Source: https://clinicaltrials.gov/.

## Data Availability

The data used to support the findings of this study have not been made available because no new data was generated.
